# Nanochannel-Controlled Synthesis of Ultrahigh Nitrogen-Doping Efficiency on Mesoporous Fe/N/C Catalysts for Oxygen Reduction Reaction

**DOI:** 10.1186/s11671-020-3254-x

**Published:** 2020-01-28

**Authors:** Chaozhong Guo, Yanrong Li, Zhaoxu Li, Yao Liu, Yujun Si, Zhongli Luo

**Affiliations:** 1 College of Materials Science and Engineering/Research Institute for New Materials Technology, Chongqing University of Arts and Sciences, Chongqing, 402160 China; 2 College of Basic Medical Sciences, Chongqing Medical University, Chongqing, 400016 China; 3 College of Chemistry and Environmental Engineering, Sichuan University of Science and Engineering, Zigong, 643000 China

**Keywords:** Fe/N/C catalyst, Iron-organic complex, Nanochannel-confined effect, Nitrogen-doped efficiency, Coordination effect

## Abstract

Designing appropriate methods to effectively enhance nitrogen-doping efficiency and active-site density is essential to boost the oxygen reduction reaction (ORR) activity of non-platinum Fe/N/C-type electrocatalysts. Here, we propose a facile and effective strategy to design a mesopore-structured Fe/N/C catalyst for the ORR with ultrahigh BET surface area and outstanding conductivity via nanochannels of molecular sieve-confined pyrolysis of Fe^2+^ ions coordinated with 2,4,6-tri(2-pyridyl)-1,3,5-triazine complexes as a novel precursor with the stable coordination effect. Combining the nanochannel-confined effect with the stable coordination effect can synergistically improve the thermal stability and stabilize the nitrogen-enriched active sites, and help to control the loss of active N atoms during pyrolysis process and to further obtain a high active-site density for enhancing the ORR activity. The as-prepared Fe/N/C electrocatalyst has exhibited excellent catalytic activity with an onset potential of ~ 0.841 V (versus RHE) closely approaching the Pt/C catalyst and high long-term stability in alkaline electrolyte. Besides, low-hydrogen peroxide yield (< 6.5%) and high electron transfer number (3.88–3.94) can be found on this catalyst, indicating that it is a valuable substitute for traditional Pt/C catalysts. This work paves a new way to design high-performance Fe/N/C electrocatalysts and deepens the understanding of active site and ORR catalysis mechanism.

## Introduction

The exploration of advanced clean energy devices (e.g., fuel cells and metal-air batteries) is currently an effective solution to solve the environmental pollution and energy crisis. In these systems, the oxygen reduction reaction (ORR) possessing the characteristics of sluggish kinetics and reaction pathway diversity is a crucial process [1, 2]. At present, the first-class and widely used electrocatalysts for the ORR are the platinum nanoparticles anchored on various carbon materials, but they meet with some disadvantages (e.g., high cost, scarcity, poor stability, and being easily poisoned) to largely limit the large-scale commercial applications [3]. Thus, design of cheap and highly efficient non-platinum or non-noble metal ORR electrocatalysts is extremely indispensable.

In recent years, the doped-carbon electrocatalysts are considered as one of potential non-noble electrocatalysts as substitutes for the commercial Pt-based catalysts in terms of these advantages such as low-cost, high-performance, corrosion-resisting, and abundant resources [4, 5]. Transition metal/nitrogen-doped carbons (TM/N/C) serving as the most important kind of doped-carbon ORR catalysts have become a popular area of research [4–6]. On the one hand, the synthesis of non-platinum TM/N/C catalysts derived from phthalocyanine, porphyrin, and their derivatives containing a TM-N_*x*_ (*x* = 2, 4, 6, et al.) structure as promising precursors [4–10], but the area of concern is commonly confined to a reason that the structure of TM-N_*x*_ is a sole prerequisite for successfully designing TM/N/C catalysts. On the other hand, some researchers proposed that the heat-treatment of metal-macrocyclic complexes at high temperatures can partially or completely destroy the original TM-N_*x*_ structure and then the effective active site structures will be regenerated [11]. It is found that the inclusion of TM-N_*x*_ coordination structures in the precursors is not a necessary condition, and almost the precursors containing TM, N, and C sources can be utilized for TM/N/C catalysts [12]. This breakthrough promotes the rational construction and performance control for various TM/N/C catalysts, especially for Fe/N/C catalysts as the most ideal production to replace the commercial Pt-based catalysts [13–15]. However, thanks to the heterogeneous structure of Fe/N/C catalysts, the identification of ORR active-sites is still controversial. There are two opinions concerning the ORR active site structures of Fe/N/C catalyst: (i) the new Fe-N_*x*_ active structures are reformed from Fe and N atoms after heat-treatment of ternary precursors at high temperatures [2, 7, 10, 13, 14]; (ii) the N-doped carbon (NC) structures derived from the carbon supports modified by nitrogen atoms during pyrolysis process, but Fe atoms are considered as the promoter to facilitate the formation of NC structures and they have little or no catalytic effect in the ORR process [16–18]. Although the role of Fe atoms has not been clearly explained in Fe/N/C catalysts, it is unambiguous that the doping of N atoms is introduced into the carbon skeleton of carbon support to boost the electrocatalytic activity.

One of concern is that the fabrication of Fe/N/C catalysts from direct carbonization of macromolecule polymers [12, 19, 20], chemical complexes and biological proteins containing Fe-N_*x*_ structures [15, 21, 22], and other iron-nitrogen sources [17, 23, 24] is commonly carried out in an open system. Besides, most of nitrogen-enriched precursors may be easy to occur at accumulation or agglomeration in carbonization process, which will prevent effective exposure of catalytically ORR active sites, promote the thermal loss of N atoms, and reduce the active-site density, resulting in the limitation of the ORR performance of Fe/N/C catalysts [25]. Another concern is that the electronic conductivity of the synthesized Fe/N/C materials also determines their ORR activity. Generally, when the high conductivity of Fe/N/C catalysts is obtained, the active nitrogen atoms will seriously lose during calcination process. In other words, the conductance and the active-site number cannot be simultaneously taken into account in an open system. To control the loss of active nitrogen atoms during pyrolysis process is still an urgent problem to enhance the electrocatalytic activity. Several research groups proposed some effective confined reaction strategies to improve this phenomenon. Some typical samples are as follows: (i) the montmorillonite with a confined interlayer space of ~ 1 nm is used as a 2D space-confined reactor to avoid the packing and the thermal loss of active nitrogen atoms and increase the conductance characteristics [26], (i) the supersaturated sodium chlorides are emerged as a totally enclosed reactor to control the nitrogen loss and increase the active site density [27], and (iii) our group recently used self-assembled 3D-NaCl aggregates as a semi-closed confined-reactor to effectively decrease the decomposition speed of active nitrogen-rich structures, leading to the increasing of the active-site density and the enhancement of the ORR activity [25]. However, these methods commonly need cumbersome pretreatment, complicate technology or limited resistance to the over-high temperature.

Here, we propose a facile and easy strategy to design a new Fe/N/C catalyst via nanochannels of molecular sieve-confined pyrolysis of Fe coordinated with 2,4,6-tri(2-pyridyl)-1,3,5-triazine complexes (Fe-TPTZ) as a novel precursor with the stable coordination effect. In carbonization process at high temperatures, this confined method can decrease the partial loss of N atoms, improve the BET surface area and mesoporous characteristics, promote the nitrogen-doping efficiency and active-site density, and increase the electronic conductivity, which can effectively boost the ORR electrocatalytic activity of Fe/N/C catalyst. The ORR kinetic behavior and catalysis mechanism of the Fe/N/C catalyst were further investigated. The excellent ORR activity with an onset potential of ~ 0.841 V (versus RHE) closely approaching the Pt/C catalyst and high long-term stability can be observed at the Fe/N/C catalyst, indicating that it is a valuable substitute for traditional Pt/C catalysts in alkaline solution. The future impact of this study provides a new idea or method for designing a high-performance non-noble Fe/N/C catalyst via integrating the stable molecular-level coordination effect and the nanochannel-confined effects and deepens the understanding of active site and ORR catalysis mechanism.

## Methods

### Synthesis of Mesoporous Fe/N/C Catalysts

**Fig. 1:**
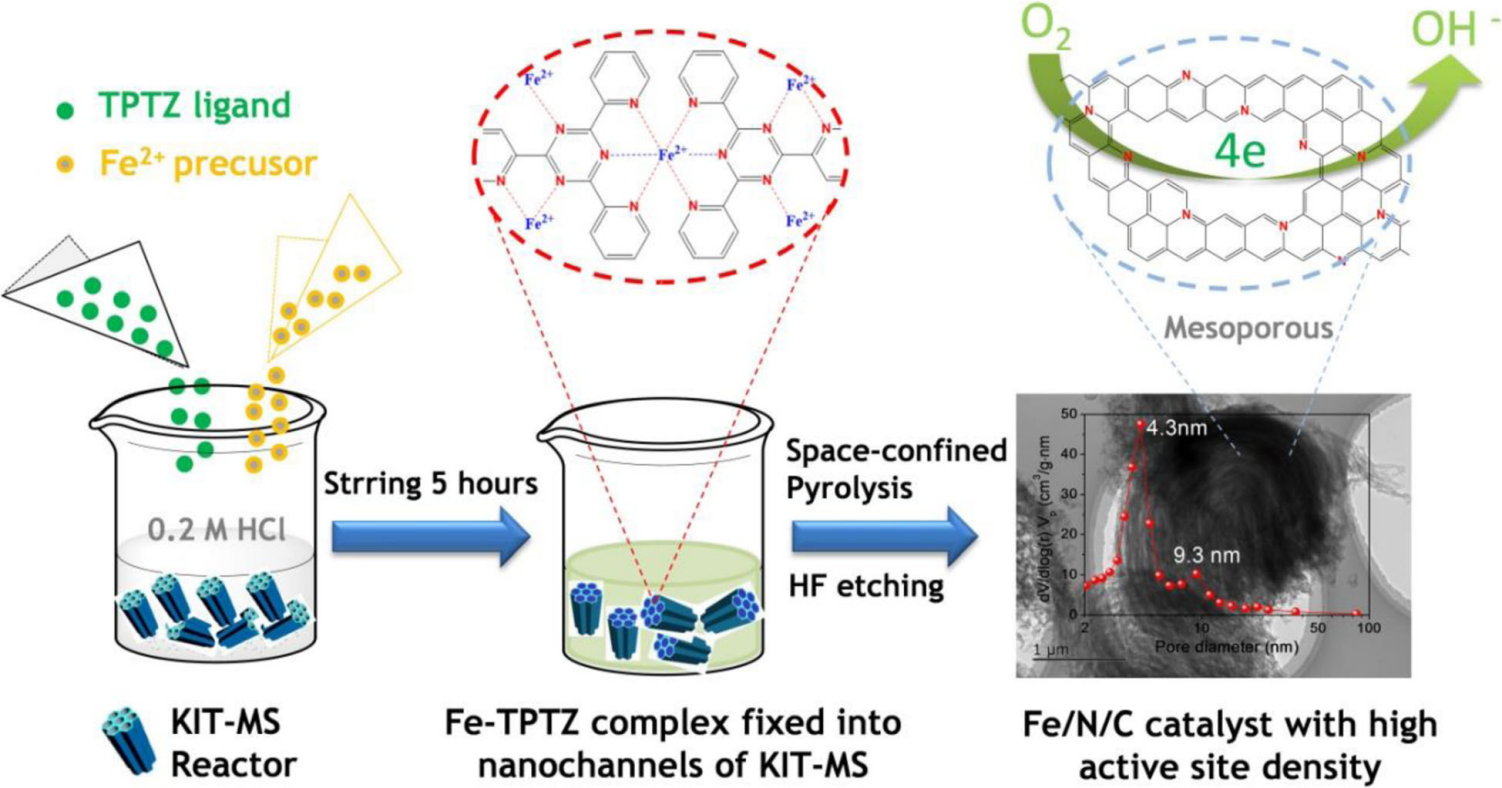
A schematic drawing for the synthesis of mesoporous Fe/N/C catalysts

In order to synthesize the Fe/N/C catalyst precursor, 0.2 g of KIT-6 molecular sieves (KIT-MS) (provided by Jiangshu Jichang Nano-Technology Co., Ltd.) with a BET surface area of 780 m^2^ g^-1^ is ultrasonically dispersed in 0.2 mol l^-1^ HCl solution (total volume 20 ml). Subsequently, 0.2 g of 2,4,6-tri(2-pyridyl)-1,3,5-triazine (TPTZ) provided by the Shanghai Aladdin Biochemical Technology Co., Ltd. and 0.0423 g of FeCl_2_·4H_2_O were successively added on the basis of a fixed condition: *n*(Fe^2+^): *n*(TPTZ) = 1: 3 and were further stirred for 5 h at 25 °C to adequately guarantee the coordination reaction between Fe^2+^ ions and the TPTZ and effectively fix the formed Fe-TPTZ complexes into nanochannels (pore size 4–10 nm) of KIT-MS. After it was dried at 80 °C in an drying oven, the gained sample is marked as the Fe-TPTZ@KIT-MS precursor, which is further heated at different temperatures (800 °C, 900 °C, and 950 °C) for 2 h in a tubular resistance furnace with a N_2_-flow rate of 0.5 L min^-1^. The yielded samples were absolutely etched by hydrofluoric acid (40 wt.%) and repeatedly washed by redistilled water to produce three kinds of mesoporous Fe/N/C catalysts (marked as *m*-Fe/N/C-800, *m*-Fe/N/C-900, and *m*-Fe/N/C-950, respectively). The designed process of mesoporous Fe/N/C catalysts with high active-site density is indicated in Fig. 1. It is notable that the KIT-MS can be considered as a new nanochannel-confined reactor for effectively controlling the thermal decomposition loss of nitrogen atoms and further improving the nitrogen-doped active-site density under the high-temperature pyrolysis process. In addition, the main reason for the Fe-TPTZ complex as a valuable starting material for production of mesopore-structured Fe/N/C electrocatalysts with high active-site density is owing to the strong coordination role between Fe^2+^ ions and nitrogen atoms in the TPTZ ligand, which can thermally stabilize the Fe-TPTZ complex structure and promote the dispersity of Fe-containing species in final Fe/N/C catalysts. Meanwhile, it can effectively decrease thermally decomposition speed of N atoms during pyrolysis process and enhance the nitrogen-doped catalytically active-site density for optimizing the ORR performance. As a control, we have prepared the *m*-Ni/N/C-900 and *m*-Cu/N/C-900 via using the same synthesis method at 900 °C and using KIT-MS as a new nanochannel-confined reactor. We also synthesized the Fe/N/C-900 and N/C-900 catalysts at the same heat-treatment temperature, derived from the Fe-TPTZ complex and TPTZ ligand, respectively, without using KIT-MS as the nanochannel-confined reactor.

### Characterization of Mesoporous Fe/N/C Catalysts

Thermogravimetric analysis data were obtained on a Shimadzu DTG-60H differential thermal analyzer under the nitrogen atmosphere with a heating speed of 10 °C min^-1^. X-ray photoelectron spectroscopy (XPS) data were collected on a Kratos XSAM800 spectrometer. High-resolution scanning and transmission electron microscopy images were gained on Hitachi UHR S4800 and FEI Tecnai-G2 F30 instruments, respectively. Nitrogen adsorption/desorption isotherms were tested on a Micromeritics ASAP 2010 analyzer at 77 K. X-ray diffraction (XRD) data were obtained on a Shimadzu XRD-6000 X-ray diffractometer (Cu Ka_1_ radiation, *λ* = 1.54178 Ǻ) at a scan rate of 4° min^-1^. Horiba HR800 Raman system was used to measure the Raman spectroscopy with an excitation wavelength of 532 nm.

### Electrochemical Tests

All electrochemical data were collected on a CHI760E Bipotentiostat (Shanghai Chenhua Instruments Co. Ltd., China) with a three-electrode system, which is composed of a rotation ring (Pt)-disk (glassy carbon, *Φ* = 5 mm) working electrode (RRDE, American Pine Instrument Co., Ltd.), a saturated calomel reference electrode (SCE) and a Pt-foil counter electrode (1 cm^2^). The preparation of the catalyst-coated RRDE refers to the previous reports [21, 25]. Generally, 10 μl of 10 mg ml^−1^ catalyst dispersion was dropped onto RRDE and naturally dried. The mass-loading of the catalyst was limited to be about 600 μg cm^−2^. All electrode potentials versus the SCE in alkaline electrolyte were converted into the potentials versus the reversible hydrogen electrode (RHE) on the basis of the Nernst equation. In other words, the potential conversion follows this equation: E (vs. RHE)/V = E (vs. SCE)/V + 1.0 V. Before doing the electrochemical test, the activation of the catalyst-coated RRDE was carried out by the cyclic voltammetry testing from 1.2 to 0.2 V vs. RHE in 0.1 mol l^-1^ KOH solution saturated by nitrogen for 20 cycles. The scanning rate is 5 mV s^-1^ and the electrolyte is 0.1 mol l^-1^ KOH in all voltammetry tests.

## Results and Discussion

**Fig. 2:**
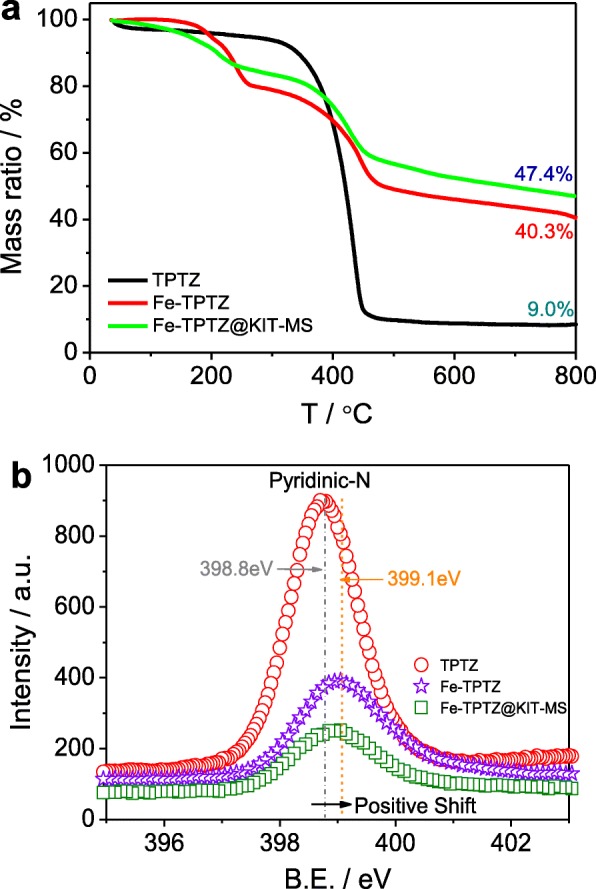
**a** TGA curves and **b** high-resolution N1*s* XPS spectra of TPTZ, Fe-TPTZ, and Fe-TPTZ@KIT-MS precursors

Thermogravimetric analysis (TGA) of three precursors (Fe-TPTZ@KIT-MS, Fe-TPTZ, and TPTZ) is first indicated in Fig. 2a. It is seen that the thermal decomposition of TPTZ ligand is fast after the temperature is beyond 300 °C and only ~ 9.0% of residual mass can be saved. However, the residual mass is ~ 40.3% at the TGA curve of Fe-TPTZ complex, suggesting the formation of the relatively stable coordination interaction of Fe^2+^ ions and nitrogen atoms such as pyridinic-nitrogen atoms on the edge or aromatic-ring nitrogen atoms in the TPTZ ligand [28]. This molecular-level complexation effect can mainly improve the thermal stability of Fe-TPTZ complex and also help bridged and cross-linked TPTZ molecules to be produced. The decomposition behavior of the Fe-TPTZ can be further retarded thanks to the usage of the space-confined role of nanochannels of KIT-MS as a novel nanoreactor, leading to the largest residual mass (47.4%) of Fe-TPTZ@KIT-MS. This role can be in favor of the increase of nitrogen-doped efficiency and active-site density during high-temperature pyrolysis, facilitating to optimize the ORR performance of mesoporous Fe/N/C catalysts. To further examine whether the stable coordination interaction was fully formed, we first characterized TPTZ, Fe-TPTZ, and Fe-TPTZ@KIT-MS by high-resolution N1s XPS spectra (Fig. 2b). Obviously, the binding energy (B.E.) of pyridinic-nitrogen shifted positively from 398.8 eV for the TPTZ ligand to 399.1 eV for the Fe-TPTZ complex or the Fe-TPTZ@KIT-MS precursor. It provides the direct evidence for the reduction of the electron density around pyridinic-N atoms after Fe^2+^ ions are introduced, because the positive shift in B.E. can be attributable to the complexation interaction that happened at between Fe^2+^ ions and pyridinic-N atoms on the TPTZ molecule edge in which 3d-unoccupied orbitals of Fe^2+^ ions can be effectively filled by the lone pair electrons of pyridinic-nitrogen atoms. The formation of the Fe-TPTZ complex can further induce more pyridinic-nitrogen atoms to be gathered around the Fe atoms, which can be beneficial to produce more Fe-N_*x*_ active site structures resulting in the ORR electrocatalytic activity enhancement of as-prepared mesopore-structured Fe/N/C catalysts.

**Fig. 3:**
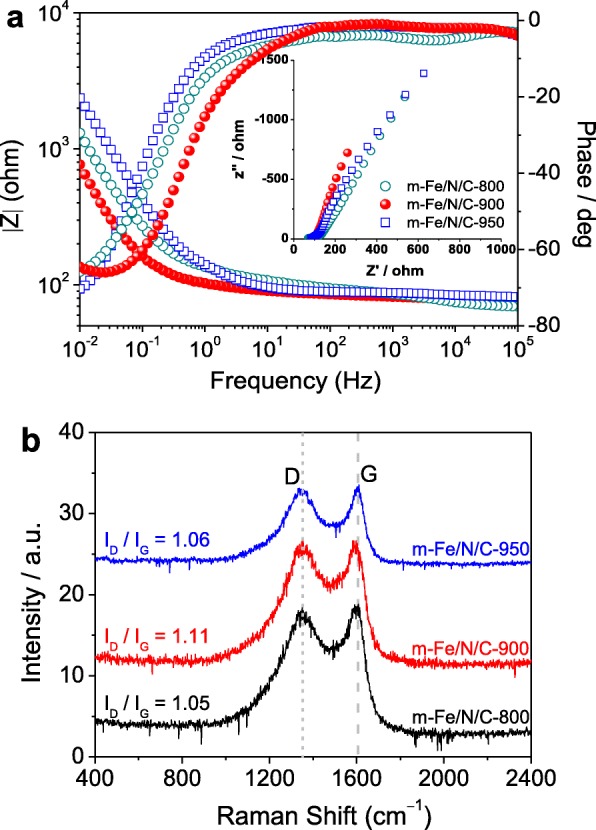
**a** EIS bode plot of m-Fe/N/C-800, m-Fe/N/C-900, and m-Fe/N/C-950; the inset is the corresponding Nyquist plot. **b** Raman spectra of m-Fe/N/C-800, m-Fe/N/C-900, and m-Fe/N/C-950

Based on that the conductance characteristic of Fe/N/C-type electrocatalysts maintained a close connection with their ORR catalytic performances, we have measured the electronic conductivity of *m*-Fe/N/C-800, *m*-Fe/N/C-900, and *m*-Fe/N/C-950 by the electrochemical impedance spectroscopy (EIS). The EIS data were obtained in a mixed solution composed of 0.1 M KCl as an electrolyte and 1 mM [Fe(CN)_6_]^3-^/[Fe(CN)_6_]^4-^ as a redox probe. The Bode results are shown in Fig. 3a, and the Nyquist plots are indicated in the inset of Fig. 3a. The Nyquist plots are fitted by an equivalent circuit (see Additional file 1: Figure S1) with five components of *R*_s_, *C*_dl_, *R*_p_, *R*_int_, and *C*_ϕ_, where *R*_s_ represents the resistance of electrolyte occurred between the reference electrode and the working electrode. *C*_dl_ represents the double-layer capacitance on the electrode solid/electrolyte interface, *C*_ϕ_ is the related relaxation of charge associated with the formation of surface intermediates, *R*_p_ represents the charge-transfer resistance during the ORR, and *R*_int_ represents the ease with the formation of intermediates. The sum of *R*_p_ and *R*_int_ is related to the ORR rate. The fitted results are suggested in Additional file 1: Table S1. It can be found that the sum of *R*_p_ and *R*_int_ is only 2232.2 Ω for *m*-Fe/N/C-900, but is about 2475.5 Ω for *m*-Fe/N/C-800 and up to 4418.6 Ω for *m*-Fe/N/C-950, respectively. A smaller sum suggests a relatively faster ORR rate, which means that the corresponding catalyst (*m*-Fe/N/C-900) has exhibited better ORR electrocatalytic activity.

Raman spectra of *m*-Fe/N/C-800, *m*-Fe/N/C-900, and *m*-Fe/N/C-950 (see Fig. 3b) were measured and then were fitted into two Lorentzian peaks. The peak centered at ~ 1350 cm^-1^ corresponds to the disordered structure-induced D-band, which is usually owing to the occurrence of various in-plane heteroatom-doped defects [18]. The peak centered at ~ 1600 cm^-1^ represents all graphitic structure-induced G-band with the in-plane *E*_2g_ vibration mode [25]. Their intensity ratio (*I*_D_/*I*_G_) was significantly utilized for discriminating the degrees of disorder and graphitization. It is found that the *I*_D_/*I*_G_ value on *m*-Fe/N/C-900 is about 1.11, which is obviously higher that on *m*-Fe/N/C-800 (*I*_D_/*I*_G_ = 1.05) or *m*-Fe/N/C-950 (*I*_D_/*I*_G_ = 1.06). It implies that more nitrogen-containing defective structures and higher doping-content of nitrogen atoms can be existed in *m*-Fe/N/C-900, which is in good accordance with the results of XPS analysis. In this study, we directly fixed the Fe-TPTZ complexes into nanochannels of molecular sieves as a new nanoconfinement reactor can significantly protect them from the pyrogenic decomposition. Besides, the strong molecular-level coordination effect between Fe^2+^ ions and nitrogen atoms in the TPTZ ligand can also stabilize the Fe-TPTZ precursor to a certain extent and further promote the total N content and N-doping efficiency during the pyrolysis process. Some previously reported results proposed that the doping content of N-atoms can positively influence the electrical conductivity of doped-carbon catalysts [22, 26], adequately supporting the optimal conductance characteristic and the highest N-doping efficiency of the *m*-Fe/N/C-900 electrocatalyst prepared in this work.

**Fig. 4:**
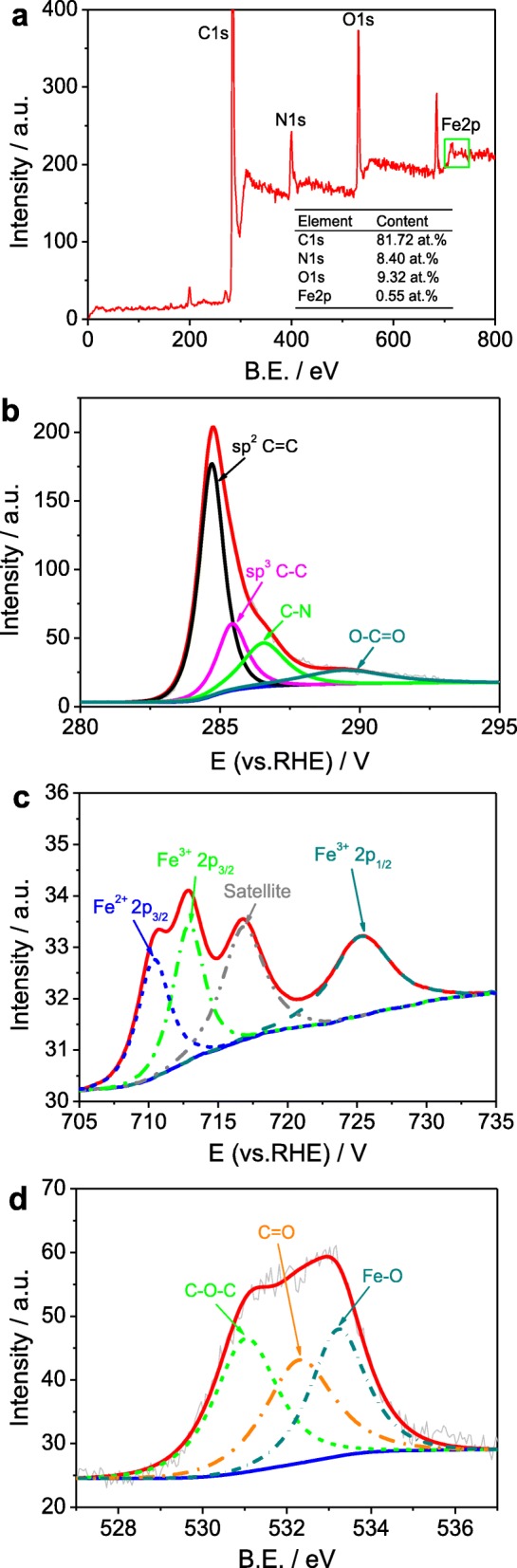
Survey (**a**), Fe 2*p* (**b**), O 1*s* (**c**), and C 1*s* (**d**) XPS spectra of *m*-Fe/N/C-900

The analysis of X-ray photoelectron spectroscopy (XPS) was applied to study the electronic structure and chemical composition on the surface of *m*-Fe/N/C-800, *m*-Fe/N/C-900, and *m*-Fe/N/C-950. According to the survey XPS spectra (Fig. 4 and Additional file 1: Figure S2), we find that all Fe/N/C-type catalysts largely contain the elements of C, N, O, and Fe, proving that Fe and N atoms were successfully doped into the carbon structure. Total contents of Fe, C, and N from the XPS surface analysis and all N/C ratios in as-prepared mesoporous Fe/N/C-type ORR electrocatalysts are summarized in Table 1. It is shown that the N/C ratio in *m*-Fe/N/C-900 is up to 10.3, which is obviously larger than that in *m*-Fe/N/C-800 (~ 10.0) or *m*-Fe/N/C-950 (~ 4.4), further suggesting the highest nitrogen-doping efficiency and total nitrogen content in *m*-Fe/N/C-900. The C1*s* XPS analysis of *m*-Fe/N/C-900 (Fig. 4b) does also approve of the doping of nitrogen atoms thanks to the appearance of a characteristic peak corresponding to the sp^2^ C=N bond at the binding energy (B.E.) of ~ 286.5 eV. Furthermore, the signal of Fe element in the survey XPS spectra of as-prepared Fe/N/C-type electrocatalysts is relatively weak because the Fe content is very low after leaching in acidic solution. To examine the electronic state of Fe atoms, we analyzed the Fe 2*p* XPS spectrum of *m*-Fe/N/C-900 in Fig. 2b, which was fitted into four peaks with B.E. of 710.3, 712.9, 716.8, and 725.3 eV, respectively. The peak located at ~ 710.3 eV can be ascribed to the characteristic peak of the Fe–N bond, showing the occurrence of the Fe^2+^ state in *m*-Fe/N/C-900 [29, 30]. The B.E. located at about 712.9 eV and 725.3 eV corresponds to the Fe^3+^ 2*p*_3/2_ and Fe^3+^ 2*p*_1/2_, respectively (Fig. 4c), which suggests that the Fe atoms from the Fe-TPTZ complex can be partially oxidized; however, the peak located at 716.8 eV can represent the satellite peak of the Fe–N bond. To further assure the chemical state of Fe oxides, we analyzed the O1*s* spectrum of *m*-Fe/N/C-900 (Fig. 4d). It is divided into three peaks with B.E. of 531.0, 532.3, and 533.2 eV, which corresponds to C–O–C, C=O, and Fe–O separately [31]. The abovementioned results may indicate that the existed state of Fe atoms is largely composed of Fe–N and Fe–O bonds in the *m*-Fe/N/C-900 electrocatalyst.

**Table 1:** Surface contents of Fe, C, and N and the N/C ratios in prepared Fe/N/C catalysts. Data derive from the XPS survey analysis

Sample	Fe content (at.%)	C content (at.%)	N content (at.%)	N/C ratio (%)
*m*-Fe/N/C-800	0.43	82.99	8.33	10.0
*m*-Fe/N/C-900	0.55	81.72	8.40	10.3
*m*-Fe/N/C-950	0.45	89.32	3.96	4.4
Fe/N/C-900	0.41	87.01	4.48	5.1

**Fig. 5:**
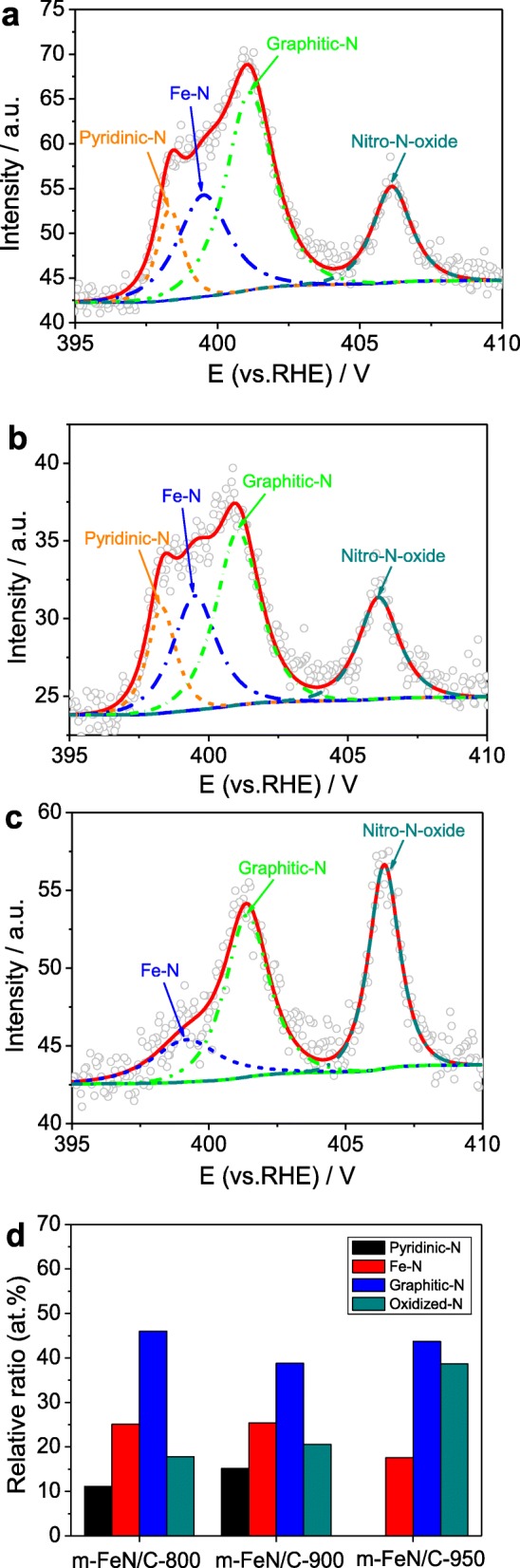
N 1*s* XPS spectra of *m*-Fe/N/C-800 (**a**), *m*-Fe/N/C-900 (**b**), *m*-Fe/N/C-950 (**c**), and their relative contents in total N content (**d**)

The fitted N1*s* XPS spectra of *m*-Fe/N/C-800 and *m*-Fe/N/C-900 (see Fig. 5a, b) display four peaks with B.E. of 398.3, 399.5, 401.1, and 406.1 eV, which are severally attributable to the pyridinic-N, Fe–N, graphitic-N, and oxidized-N (–NO_2_) [10, 16, 25, 26, 32, 33]. However, the fitted N1*s* XPS spectrum of *m*-Fe/N/C-950 (Fig. 5c) has showed only three peaks with B.E. of 399.2, 401.4, and 406.4 eV, corresponding to the Fe–N, graphitic-N, and oxidized-N (–NO_2_), respectively. It is noted that the electron cloud density of nitrogen atoms is shifted due to a stronger electronegativity of oxygen atoms, resulting in the oxidation peak appeared at the high energy stage. The relative ratio of graphitic-N groups dominates in the total nitrogen content, but the relative ratio of inactive oxidized-N (–NO_2_) increases from 17.8 to 38.7 at.% with an increment of the pyrolysis temperature, as indicated in Fig. 5d. Besides, higher pyrolysis temperature will lead to the disappearance of pyridinic-N group in *m*-Fe/N/C-950. Above results show that the Fe–N group can be formed in as-prepared Fe/N/C-type catalysts, but active pyridinic- and graphitic-N groups are of great significance to determine the ORR activity of Fe/N/C-type electrocatalysts.

**Fig. 6:**
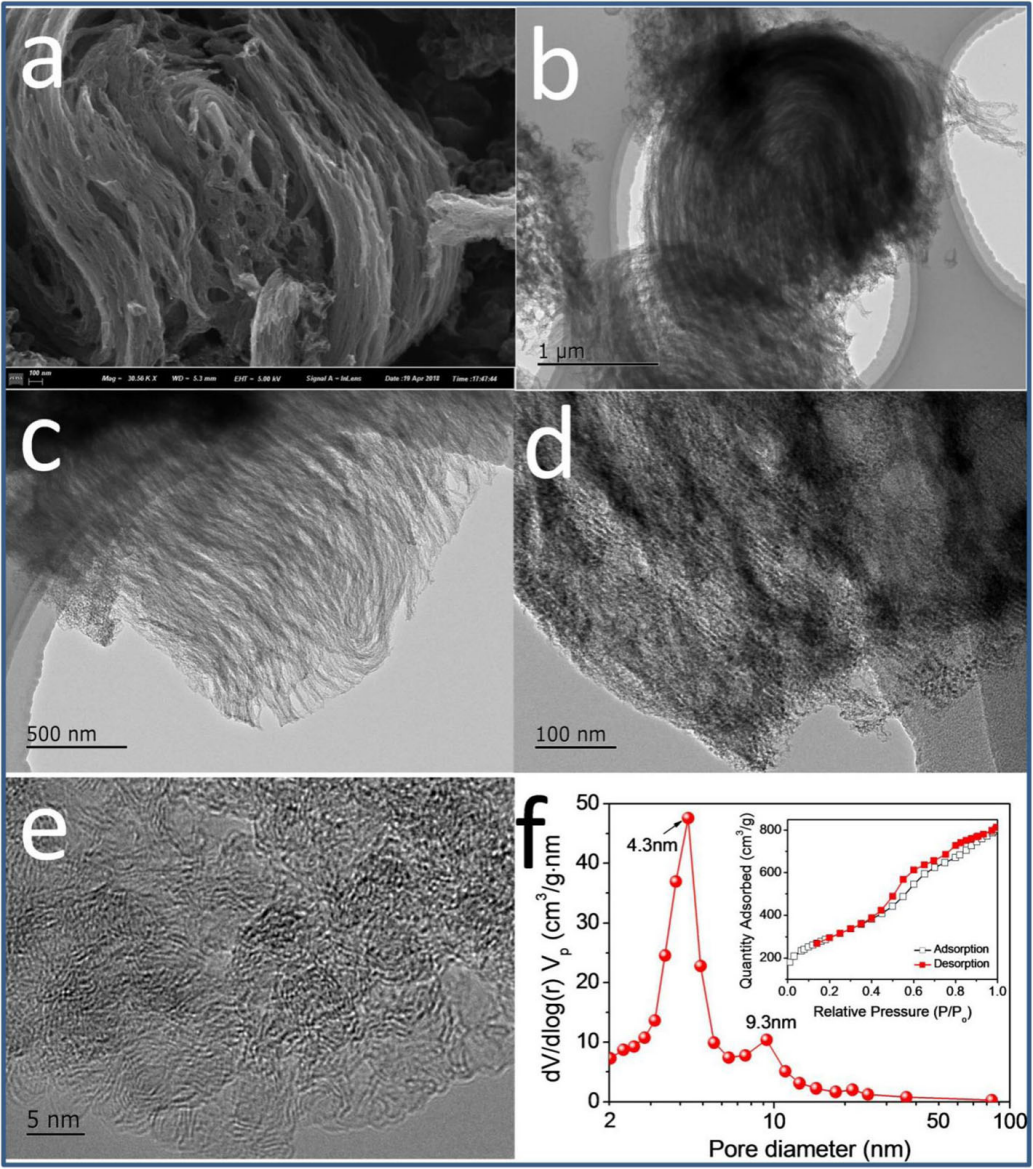
SEM (**a**) and high-resolution TEM images (**b**–**e**) of *m*-Fe/N/C-900; **f** the pore-size distribution of *m*-Fe/N/C-900; the inset is nitrogen adsorption-desorption isotherms

**Fig. 7:**
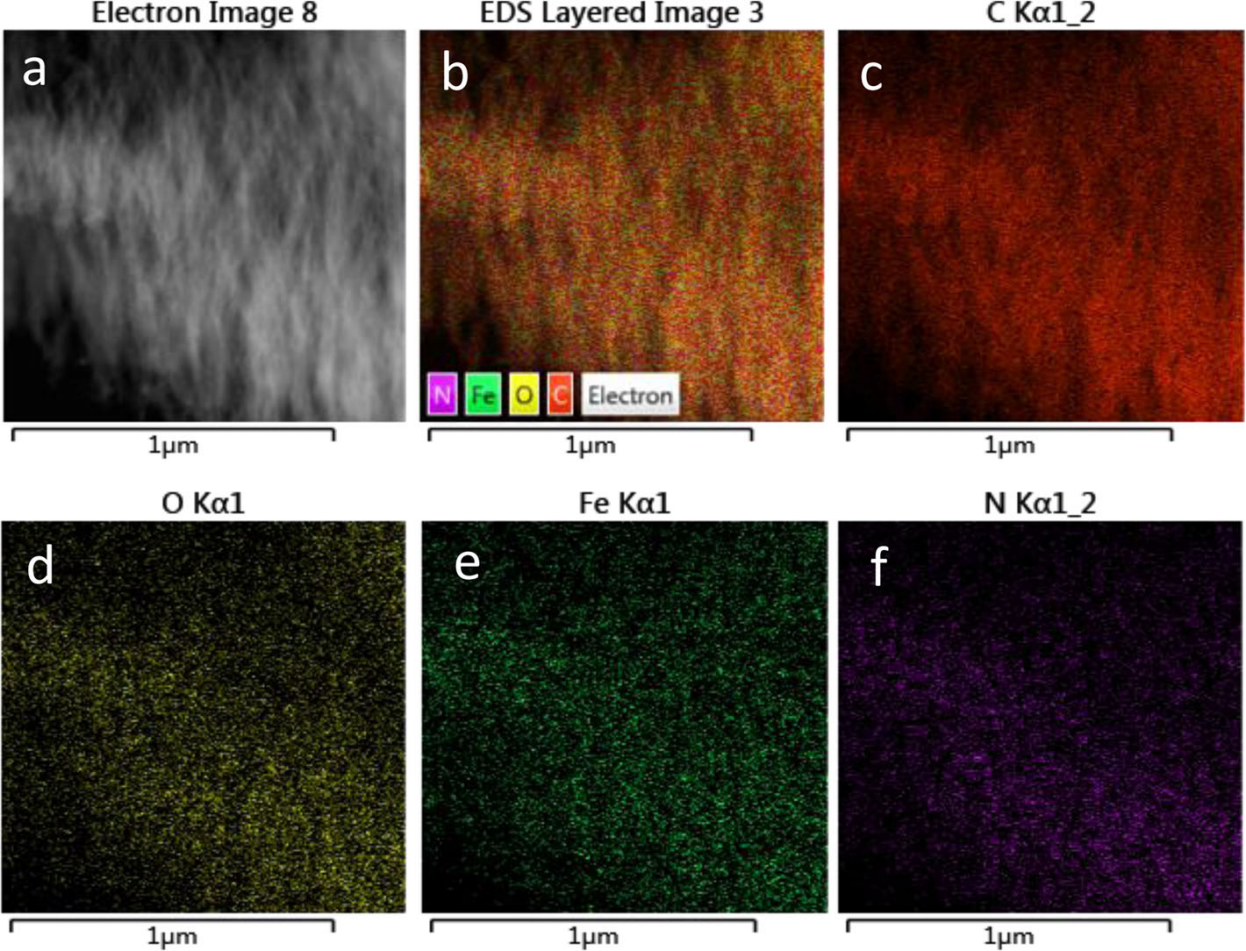
TEM image (**a**) of *m*-Fe/N/C-900 and corresponding elemental mapping images of C, O, Fe, and N (**b**–**f**)

The morphology analysis of *m*-Fe/N/C-900 catalyst was depicted in Fig. 6 and Additional file 1: Figure S3. A large number of spongy-like shapes can be existed for *m*-Fe/N/C-900 (Fig. 6a, b) synthesized by high-temperature pyrolysis of metal-organic (Fe-TPTZ) complexes confinedly fixed into nanochannels of molecular sieves. High-resolution TEM images (Fig. 6c, d) significantly displays that there are numerous highly ordered mesoporous structures inside *m*-Fe/N/C-900, which can largely derive from the removal of molecular sieve as a nanochannel-confined reactor. Besides, the disordered carbon structures on the edge and several mesopores can be clearly seen in Fig. 6e, which is owing to the doping of nitrogen atoms. For this reason, porous characteristic and Brunauer-Emmett-Teller (BET) surface area of *m*-Fe/N/C-900 were also studied by nitrogen adsorption/desorption isotherms (the inset of Fig. 6f). A Langmuir IV-type isotherm curve can be observed, suggesting the highly mesoporous characteristic of the *m*-Fe/N/C-900 electrocatalyst. It can be further confirmed by the BJH pore-size distribution of *m*-Fe/N/C-900 (Fig. 6f), which has displayed a high BET surface area (*A*_BET_ ~ 1035 m^2^ g^-1^) and total pore volume (*V*_total_ ~ 1.22 cm^3^ g^-1^) with an average pore-diameter (*D*_p_) of about 4.7 nm. The exhibited two maximum pore-sizes (4.3 and 9.3 nm) are ascribed to the maximum position of mesopores in *m*-Fe/N/C-900. These mesopores can provide more convenient nanochannels for fast transportation of the electrolyte, reactants, and products; decrease the transportation resistance of the oxygen molecule to the nitrogen-doped active sites; and boost the ORR catalytic performance of *m*-Fe/N/C-900. In addition, the use of nanochannels of molecular sieve as a nanoconfined reactor is beneficial to produce spongy-like three-dimensional mesoporous carbons with ultrahigh surface area, facilitating to enhance the exposure of catalytically ORR-active sites. The elemental mapping images (Fig. 7) display the homogeneous distribution of four kinds of main elements (Fe, N, C, and O) on the surface of *m*-Fe/N/C-900, which may be resulted from the synergistic roles of the nanochannel-confined effect of molecular sieves and the stable molecular-coordination effect of Fe-TPTZ complexes to a certain extent.

**Fig. 8:**
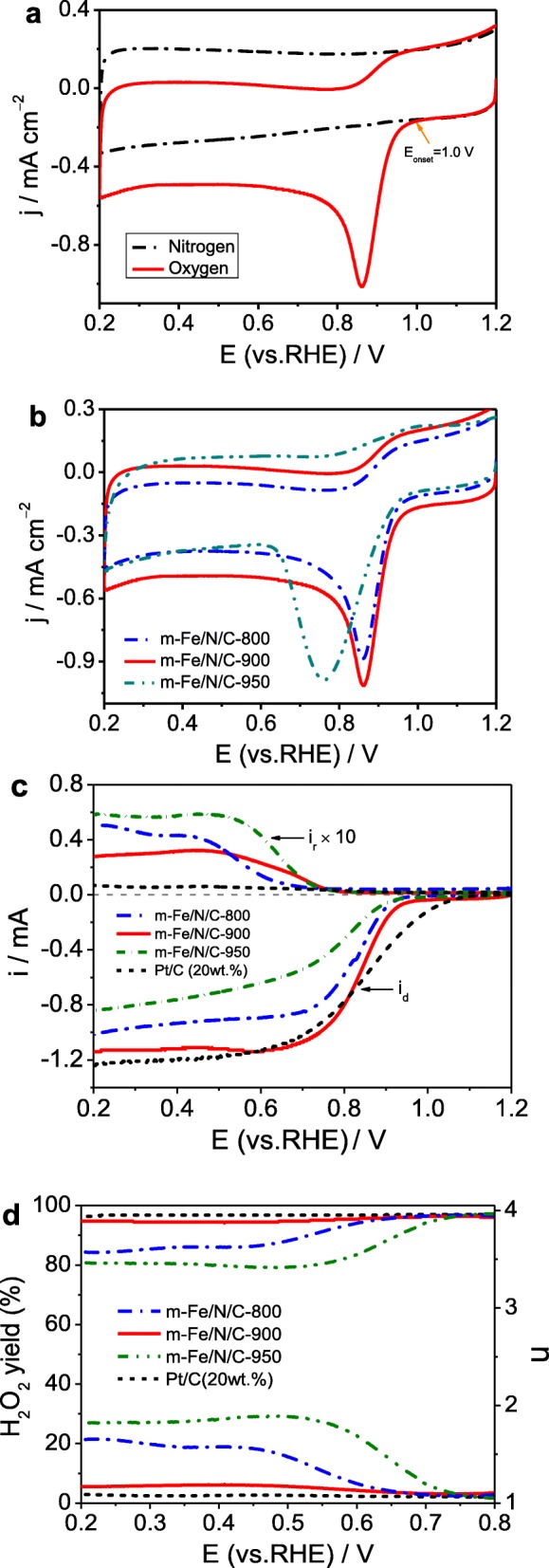
**a** CV curves of *m*-Fe/N/C-900 in N_2_ vs O_2_-saturated 0.1 M KOH solution. **b** CV curves of *m*-Fe/N/C-800, *m*-Fe/N/C-900, and *m*-Fe/N/C-950 in O_2_-saturated 0.1 mol l^-1^ KOH solution. **c** Disk and ring currents obtained with LSVs on RRDE for *m*-Fe/N/C-800, *m*-Fe/N/C-900, and *m*-Fe/N/C-950 in O_2_-saturated 0.1 mol l^-1^ KOH solution. **d** The corresponding electron transfer numbers and H_2_O_2_ yields of *m*-Fe/N/C-800, *m*-Fe/N/C-900, and *m*-Fe/N/C-950 derived from **c**

The electrocatalytic activities towards the ORR of all prepared catalysts were tested by the cyclic voltammogram (CV) or linear scanning voltammogram (LSV). Figure 8a shows CVs of *m*-Fe/N/C-900 in N_2_ versus O_2_-saturated 0.1 mol l^-1^ KOH electrolyte. A sharp ORR peak occurred at 0.86 V vs. RHE in O_2_-saturated electrolyte; however, a featureless CV curve was observed in N_2_-saturated electrolyte, suggesting the ORR catalytic activity of *m*-Fe/N/C-900 with an onset potential (*E*_onset_) of 1.0 V. Besides, the effect of the heat-treatment temperature (800–950 °C) on the ORR performance was investigated in Fig. 8b. Higher or lower heat-treatment temperature make against the activity enhancement based on that the largest peak current density (*j*_p_), and the most positive peak potential (*E*_p_) can be obtained at *m*-Fe/N/C-900. The ORR activity of *m*-Fe/N/C-900 can be comparable to other reported doped-carbon catalysts (see Additional file 1: Table S2). To get insights into the ORR kinetic behavior of Fe/N/C-type electrocatalysts, we further tested the ORR polarization curves by LSV method combined with the RRDE, as indicated in Fig. 8c. On the basis of the RRDE data, the transferred electron number (*n*) and H_2_O_2_ yield (H_2_O_2_%) during the ORR were estimated via using the following Eqs. (1) and (2), respectively. The calculated equations are as follows [34]:

1 $$ \%H{O}_2^{-}=100\times \frac{2{I}_r/N}{I_d+\left({I}_r/N\right)} $$

2 $$ n=4\times \frac{I_d}{I_d+{I}_r/N} $$

where *I*_d_ is the Faradaic disk-current, *I*_r_ is the Faradaic ring-current, and *N* is the collection efficiency of ring electrode (0.38). The Pt-ring potential was set at 1.5 V (vs*.* RHE) as reported elsewhere. Figure 8d displays the relatively calculated results. The H_2_O_2_ yield (< 6.5%) and *n* value (3.88–3.94) are obtained on *m*-Fe/N/C-900, dominating a four-electron ORR pathway. It suggests that this catalyst is a valuable substitute for the traditional 20 wt.% Pt/C catalyst (purchased from Aladdin Industrial Co. Ltd.), although the H_2_O_2_ yield on *m*-Fe/N/C-900 is slightly higher. In addition, the half-wave potential (*E*_1/2_) for ORR of *m*-Fe/N/C-900 is about 0.841 V approaching that of 20 wt.% Pt/C (~ 0.848 V), and the limited current density (*j*_d_) of *m*-Fe/N/C-900 is almost identical to that of 20 wt.% Pt/C. Compared to the *m*-Fe/N/C-900 catalyst, higher H_2_O_2_ yield and smaller *n* value can be gained on both *m*-Fe/N/C-800 and *m*-Fe/N/C-950, but the electron transfer number on both *m*-Fe/N/C-800 and *m*-Fe/N/C-950 still belongs to the range (3.4–4.0), showing that the ORR on two Fe/N/C-type happens with a mixed process of two and four-electron transfer pathways. The above results further approve that the *m*-Fe/N/C-900 synthesized by nanochannel-confined control of the pyrolysis process to improve the nitrogen-doping efficiency and increase the nitrogen-doped active-site density has exhibited the optimal ORR catalytic performance in alkaline electrolyte.

**Fig. 9:**
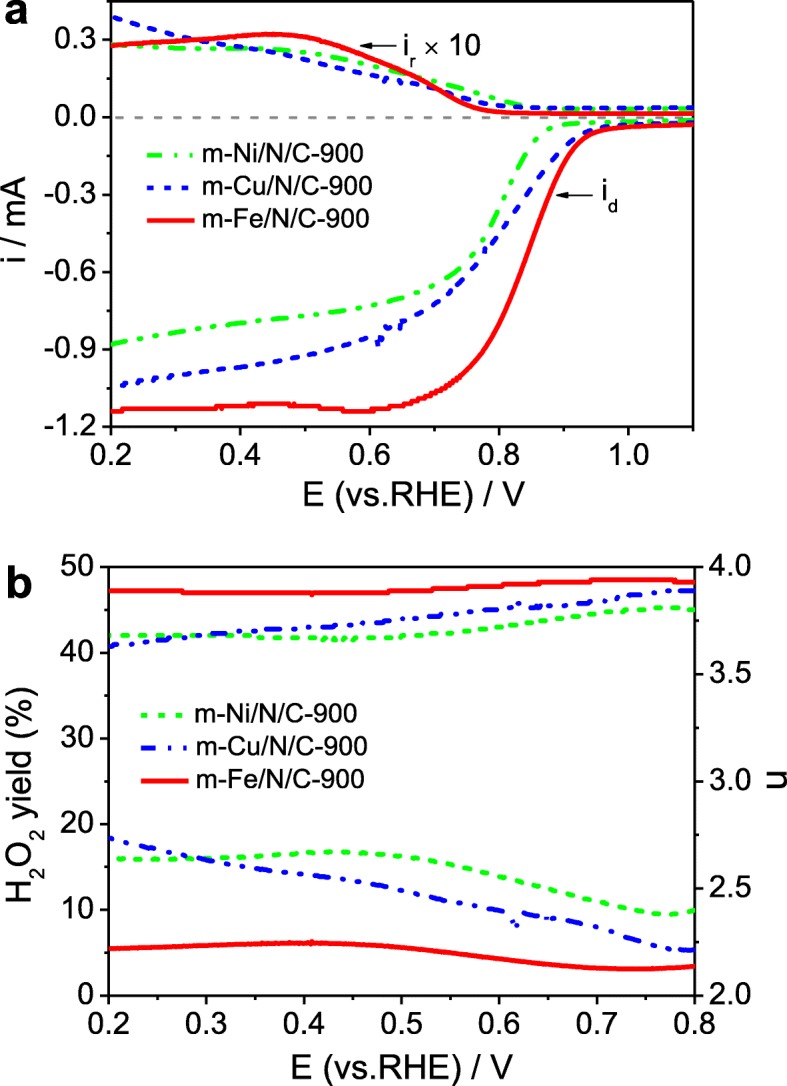
**a** Disk and ring currents obtained with LSVs on RRDE for *m*-Ni/N/C-900, *m*-Cu/N/C-900, and *m*-Fe/N/C-900 in O_2_-saturated 0.1 mol l^-1^ KOH solution. **b** The corresponding electron transfer number and H_2_O_2_ yield of *m*-Ni/N/C-900, *m*-Cu/N/C-900, and *m*-Fe/N/C-900 derived from **a**. **c** CV curves and **d** LSV curves of *m*-Fe/N/C-900 before and after continuous scanning for 5000 cycles in O_2_-saturated 0.1 M KOH solution

We also discuss the effect of different transition metals on the ORR catalytic activity of Fe/N/C-type electrocatalysts. The obtained LSV curves on the RRDE are indicated in Fig. 9a, and the corresponding *n* value and H_2_O_2_ yield are demonstrated in Fig. 9b. The *E*_1/2_ values are about 0.785 V for *m*-Cu/N/C-900 and 0.780 V for *m*-Ni/N/C-900, respectively, which are lower compared to the *m*-Fe/N/C-900. The *j*_d_ follows the order of *m*-Fe/N/C-900 > *m*-Cu/N/C-900 > *m*-Ni/N/C-900, further suggesting the best ORR catalytic activity of *m*-Fe/N/C-900 in 0.1 mol l^-1^ KOH solution. Compared to the *m*-Fe/N/C-900 catalyst, higher H_2_O_2_ yield and smaller *n* value are obtained on both *m*-Cu/N/C-900 and *m*-Ni/N/C-900. What is noteworthy is that the H_2_O_2_ yield on *m*-Cu/N/C-900 and *m*-Ni/N/C-900 is over twice as large as that on *m*-Fe/N/C-900. However, the *n* value on both *m*-Cu/N/C-900 and *m*-Ni/N/C-900 is 3.5–4.0, indicating that the ORR process on two Fe/N/C-type electrocatalysts follows a two- and four-electron mixed transfer pathway but is dominant in a four-electron reaction pathway. Besides, the electrochemical long-term stability for ORR catalysis of *m*-Fe/N/C-900 is of great significance in the practical applications. An accelerated aging test (AAT) was carried out by successive CV scanning tests from 0.2 to 1.2 V vs RHE for 5000 cycles at 200 mV s^-1^ in oxygen-saturated 0.1 M KOH electrolyte. The ORR electrocatalytic behavior of *m*-Fe/N/C-900 has been further evaluated under the same conditions as above experiments. CV curves for ORR activity of *m*-Fe/N/C-900 before and after doing the AAT are almost unchanged on the *E*_p_ (~ 0.86 V), but the *j*_p_ is slightly reduced (Fig. 9c). LSV curves of *m*-Fe/N/C-900 (Fig. 9d) also reveal an only ~ 12 mV negative shift in the *E*_1/2_ and a negligible decrease in the *j*_d_. However, the commercial Pt/C (20 wt.% Pt) catalyst after doing the AAT has exhibited about 55 mV of the negative shift in *E*_1/2_ and an obvious reduction in the *j*_d_ (Fig. 9d). Results show that the *m*-Fe/N/C-900 has more excellent electrocatalytic stability compared to the Pt/C catalyst, further suggesting that it is a valuable and promising substitute for the conventional Pt-based materials in alkaline electrolytes.

**Fig. 10:**
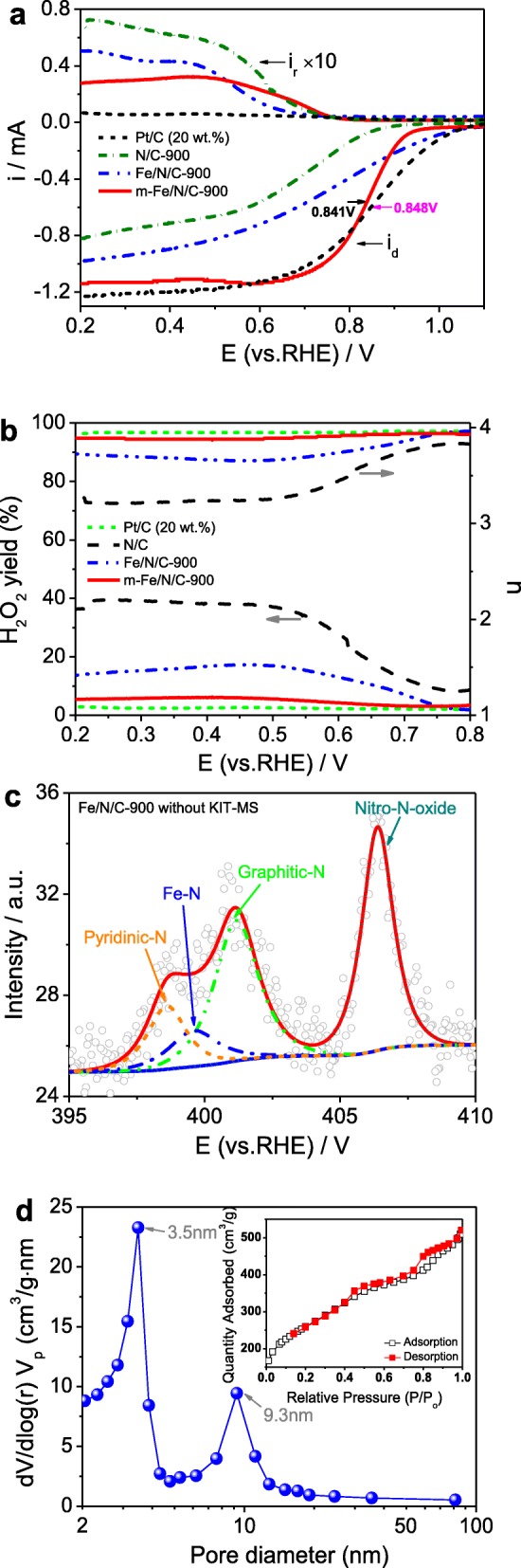
**a** Disk and ring currents obtained with LSVs on RRDE for N/C-900, Fe/N/C-900, *m*-Fe/N/C-900, and Pt/C (20 wt.%) in O_2_-saturated 0.1 mol l^-1^ KOH solution. **b** The corresponding electron transfer number and H_2_O_2_ yield of N/C-900, Fe/N/C-900 *m*-Fe/N/C-900, and Pt/C (20 wt.%) derived from **a**

In order to discuss the catalytically active sites of Fe/N/C-type catalysts and study the role of the molecular coordination and nanochannel-confined effects, we have further examined the N/C-900, Fe/N/C-900, and 20 wt.% Pt/C catalysts for comparison of the ORR catalytic behavior. The tested results of the ORR activity are indicated in Fig. 10a. The onset potentials of ORR are about 0.683 V for N/C-900 and 0.740 V for Fe/N/C-900, being largely lower than those of *m*-Fe/N/C-900 (0.841 V) and 20 wt.% Pt/C catalysts (0.848 V). Figure 10b shows the corresponding H_2_O_2_ yields and transferred electron numbers in the ORR process. Given other Fe/N/C-type catalysts and the Pt/C catalyst, the H_2_O_2_ yield on N/C-900 is the highest and the transferred electron number on N/C-900 is the smallest, suggesting the worst ORR catalytic activity of N/C-900. In addition, the H_2_O_2_ yield on Fe/N/C-900 is mainly higher than that on *m*-Fe/N/C-900 and the transferred electron number on Fe/N/C-900 is far lower than that on *m*-Fe/N/C-900 in the same range (0.2–0.8 V vs RHE), indicating a relatively inferior ORR activity. Thus, it can be concluded that the ORR performance complies with the sequence of Pt/C > *m*-Fe/N/C-900 > Fe/N/C-900 > N/C-900. These results show that the formation of Fe-TPTZ compounds with the strong molecular-level coordination effect is beneficial to produce the Fe/N/C-type catalysts with high ORR activity, and the utilization of the nanochannel-confined effect can reduce the decomposition speed of Fe-TPTZ compounds and protect the nitrogen-rich active sites (e.g., Fe–N, graphitic-N, or pyridinic-N) from the thermal loss during the pyrolysis process (see the TG analysis, Fig. 2a), which can enhance the ORR performance of Fe/N/C-type catalysts in alkaline medium.

To deepen the understanding of active sites and their ORR catalysis mechanism, we also characterized the prepared Fe/N/C-type catalysts by the spectra of X-ray diffraction (XRD) and X-ray photoelectric spectroscopy (see Additional file 1: Figures S4 and S5). The XRD data display that the density of carbon (002) peak follows the sequence of N/C-900 > Fe/N/C-900 > *m*-Fe/N/C-900, and the positions for carbon (002) peak in three catalysts are negatively shifted because of the production of more sp^2^ C–N groups into the graphitic layers and the decrease of graphitization. It also implies that the different N content may be doped into the carbon skeleton in the catalyst, and both N-doping efficiency and N content can follow the similar order with their ORR activity. More significantly, we further compare the structural and porous differences between Fe/N/C-900 and *m*-Fe/N/C-900 to better study the nanochannel-confined effect of molecular sieves. The tested XPS survey spectrum of Fe/N/C-900 was indicated in Additional file 1: Figure S5, and its surface contents of Fe, C, and N and the N/C ratio were summarized in Table 1. The Fe content and N content are ~ 0.41 at.% and ~ 4.48 at.%, but the N/C ratio is only 5.1 in the Fe/N/C-900, being lower compared to the *m*-Fe/N/C-900. It suggests the N-doping efficiency was improved by introducing the nanochannel-confined protection strategy into the carbonization process, which can prove our key views of this work. Besides, the fitted N1*s* XPS spectrum of Fe/N/C-900 is indicated in Fig. 10c. It displays the existence of four peaks with B.E. of 398.6, 399.6, 401.2, and 406.4 eV, which still correspond to the pyridinic-N, Fe–N, graphitic-N, and oxidized-N (–NO_2_) with a relative percentage of 14.0, 10.2, 35.4, and 40.4 at.%, respectively. Compared with the *m*-Fe/N/C-900, the total ratio of active N-rich groups such as pyridinic-N, Fe–N, and graphitic-N obviously decreases about 19.8 at.%; however, the relative ratio of Fe–N groups is reduced about 15.2 at.%. Thus, associating with fore-mentioned XPS data and the catalytic activity data, we conclude that the electrocatalytically active sites may be pyridinic- and graphitic-N groups for holding the ORR performance, but the enhancement of the ORR activity may be related to the relative ratio of Fe–N groups for our system. The role of the nanochannel-confined effect cannot only reduce the loss of total N content, but also can largely increase the N-doping efficiency and improve the effective ORR active-site density in the catalyst. Besides, we further analyzed porous characteristic and BET specific surface area of Fe/N/C-900 without the usage of the KIT-MS nanoreactor. The nitrogen adsorption/desorption isotherms with a similar Langmuir IV-type isotherm curve are seen in the inset of Fig. 10d. It suggests that highly mesoporous characteristic is still existed in Fe/N/C-900, supported by the analysis of BJH pore-size distribution (Fig. 10d). The *A*_BET_ (~ 875 m^2^ g^-1^) and *V*_total_ (~ 0.76 cm^3^ g^-1^) with an average *D*_P_ of only ~ 3.5 nm are obtained on the Fe/N/C-900, which are obviously lower than those on the *m*-Fe/N/C-900. The large difference on pore structures between Fe/N/C-900 and *m*-Fe/N/C-900 can be derived from the stable coordination effect and the nanochannel-confined role of a KIT-MS reactor in the preparation of *m*-Fe/N/C-900. It will also influence their inherent ORR performance because higher *A*_BET_ and *V*_total_ can help to supply abundant catalytic sites and increase the exposed surface active-site density, being beneficial to the adsorption and electro-reduction process of O_2_ molecule [35]. Notably, we should pay much attention to the effect of the conductivity characteristic. Generally, a higher conductivity characteristic of *m*-Fe/N/C-900 corresponds to a relatively faster ORR electron transportation process. Therefore, facile design and control of active nitrogen-rich groups (pyridinic-N, graphitic-N, and Fe–N, etc.) is of great importance to fabricate mesopore-structured Fe/N/C electrocatalysts for the ORR, but further improving the conductance, N-doped active-site density, and mesoporous characteristic is another key issue of concern to obtain the high performance.

## Conclusions

In conclusion, here, we propose a new and effective strategy to design a Fe/N/C-type electrocatalyst (*m*-Fe/N/C-900) with ultrahigh BET surface area (1035 m^2^ g^-1^) and total pore volume (1.22 cm^3^ g^-1^) via nanochannel-confined high-temperature carbonization of Fe^2+^ ions coordinated with 2,4,6-tri(2-pyridyl)-1,3,5-triazine compound as a single-source Fe, N, and C precursor. The elemental mapping images of *m*-Fe/N/C-900 further prove the homogeneous distribution of Fe, N, C, and O elements on its surface. On the one hand, the strong molecular-coordination role in Fe-TPTZ complex can enhance the thermal stability and stabilize higher contents of Fe–N active sites during pyrolysis process. On the other hand, the utilization of abundant nanochannels of molecular sieve as a novel nanoconfined reactor does not only benefit to produce spongy-like mesoporous carbons with excellent pore structure and conductivity characteristic, but also facilitate to decrease the loss of N atoms and improve the N-doping efficiency and N-doped active-site density, resulting in the ORR activity enhancement. Electrochemical tests indicate the *m*-Fe/N/C-900 displays unexpected catalytic performance with an ORR half-wave potential of ~ 0.841 V versus RHE and high limited current density approaching the commercial Pt/C catalyst. Additionally, low H_2_O_2_ yield (< 6.5%) and high electron transfer number (3.88–3.94) on *m*-Fe/N/C-900, indicating that it is a valuable substitute for the traditional Pt/C catalyst. The comparison analysis of XPS data and electrocatalytic activity data can point out that active pyridinic and graphitic-N groups may be the electrocatalytically ORR-active sites, but the enhancement of the ORR activity may be related to the relative ratio of Fe–N groups for our system. This study provides a new idea or method for the synthesis of high-performance Fe/N/C electrocatalysts via integrating molecular-level coordination and nanochannel-confined effects and does also help the researchers better deepen the understanding of nitrogen-doped active sites and their ORR catalysis mechanism for Fe/N/C-type electrocatalysts to a certain extent. However, what cannot be ignored is that effective improvement and optimization of nitrogen-doping active site density, conductivity, and porous characteristics is essential to boost the ORR electrocatalytic activity.

## Supplementary information

**Additional file 1: Figure S1.** The equivalent circuit of the Nyquist plots. **Figure S2.** Survey XPS spectrum of m-Fe/N/C-800 (a) and m-Fe/N/C-950 (b). **Figure S3.** High-resolution SEM image of m-Fe/N/C-900. **Figure S4.** XRD patterns of N/C-900, Fe/N/C-900 and m-Fe/N/C-900. **Figure S5.** Survey XPS spectrum of Fe/N/C-900. **Table S1**. EIS parameters for Nyquist plots of *m*-Fe/N/C-800, *m*-Fe/N/C-900 and *m*-Fe/N/C-950. **Table S2.** Comparison of this result with other reported references on the ORR activity.

## Data Availability

The authors declare that the materials and datasets used or analyzed during the current study are available from the corresponding author on reasonable request.

## Abbreviations

AAT: Accelerated aging test
AE: Auxiliary electrode
BET: Brunauer-Emmett-Teller
CV: Cyclic voltammetry
*E*_1/2_: Half-wave potential
*E*_p_: Peak potential
Fe/N/C catalyst: Iron/nitrogen/carbon catalyst
Fe-TPTZ: Fe coordinated with 2,4,6-tri(2-pyridyl)-1,3,5-triazine complexes
GC: Glassy carbon
HR-TEM: High-resolution transmission electron microscopy
KIT-MS: KIT-6 molecular sieves
LSV: Linear sweep voltammetry
ORR: Oxygen reduction reaction
Pt/C: Platinum/carbon catalyst
RDE: Rotation disk electrode
RE: Reference electrode
RHE: Reversible hydrogen electrode
RRDE: Rotation ring-disk electrode
SCE: Saturated calomel electrode
SEM: Scanning electron microscopy
TGA: Thermogravimetric analysis
TPTZ: 2,4,6-Tri(2-pyridyl)-1,3,5-triazine
WE: Working electrode
XPS: X-ray photoelectron spectroscopy
XRD: X-ray diffraction
