# Supplementary material for: Nanochannel-Controlled Synthesis of Ultrahigh Nitrogen-Doping Efficiency on Mesoporous Fe/N/C Catalysts for Oxygen Reduction Reaction
Source: Nanoscale Res Lett. 2020 Jan 28;15:21. doi: 10.1186/s11671-020-3254-x (PMC6987278; doi:10.1186/s11671-020-3254-x)
Supplement: Supplementary file 1 — Additional file 1: Figure S1. The equivalent circuit of the Nyquist plots. Figure S2. Survey XPS spectrum of m-Fe/N/C-800 (a) and m-Fe/N/C-950 (b). Figure S3. High-resolution SEM image of m-Fe/N/C-900. Figure S4. XRD patterns of N/C-900, Fe/N/C-900 and m-Fe/N/C-900. Figure S5. Survey XPS spectrum of Fe/N/C-900. Table S1. EIS parameters for Nyquist plots of m-Fe/N/C-800, m-Fe/N/C-900 and m-Fe/N/C-950. Table S2. Comparison of this result with other reported references on the ORR activity. [file 11671_2020_3254_MOESM1_ESM.docx]

**Supporting Information**

**Nanochannels-Controlled Synthesis of Ultrahigh Nitrogen-Doping Efficiency on Mesoporous Fe/N/C Catalysts for Oxygen Reduction Reaction**

Chaozhong Guo^a*^, Yanrong Li^a^, Zhaoxu Li^b^, Yao Liu^a^, Yujun Si^c*^, Zhongli Luo^b*^

^a^ College of Materials Science and Engineering/Research Institute for New Materials Technology, Chongqing University of Arts and Sciences, Chongqing 402160, China.

^b^ College of Basic Medical Sciences, Chongqing Medical University, Chongqing 400016, China.

^c^ College of Chemistry and Environmental Engineering, Sichuan University of Science and Engineering, Zigong, 643000, China.

**Corresponding authors.** E-mail: guochaozhong1987@163.com (C. Guo); [syj08448@163.com (Y. Si);](mailto:guoqiangli1989@126.com;) [zhongliluo@163.com](mailto:zhongliluo@163.com) (Z. Li)

**Figure 1S.** The equivalent circuit of the Nyquist plots.

**Figure 2S.** Survey XPS spectrum of m-Fe/N/C-800 (a) and m-Fe/N/C-950 (b).


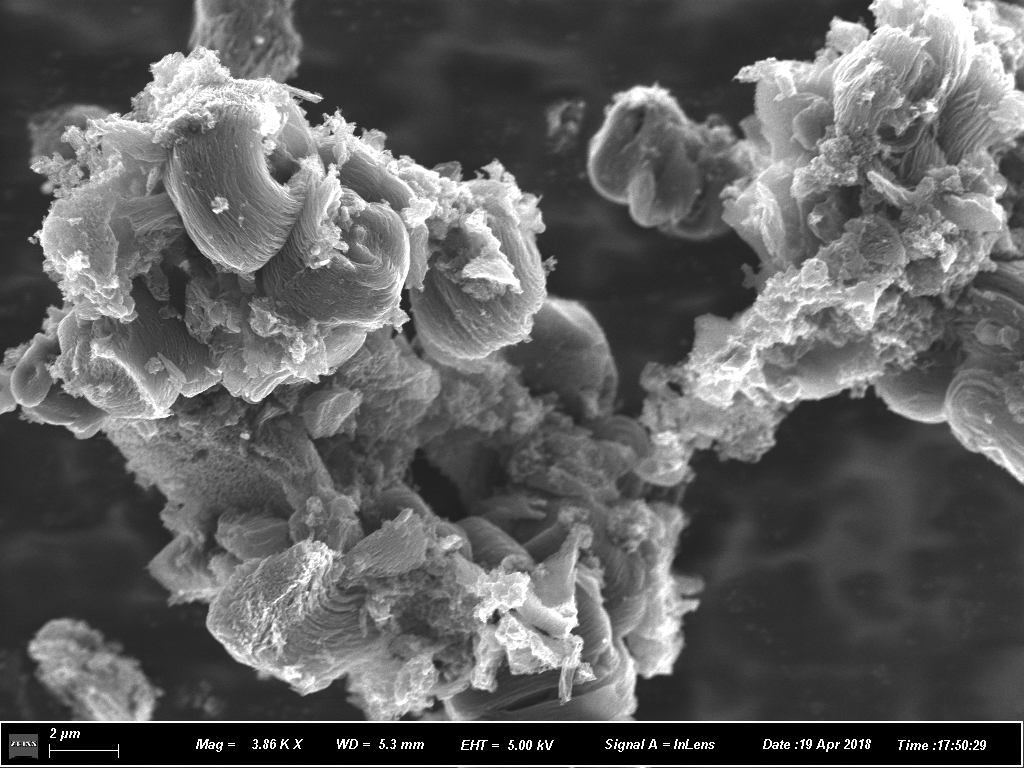


**Figure 3S.** High-resolution SEM image of m-Fe/N/C-900.

**Figure 4S.** XRD patterns of N/C-900, Fe/N/C-900 and m-Fe/N/C-900.

**Figure 5S.** Survey XPS spectrum of Fe/N/C-900.

**Table 1S**. EIS parameters for Nyquist plots of *m*-Fe/N/C-800, *m*-Fe/N/C-900 and *m*-Fe/N/C-950.

| Sample | R_s_ / Ω | R_p_ / Ω | R_int_ / Ω | C_dl_ / mF | C_ϕ_ / mF |
| --- | --- | --- | --- | --- | --- |
| *m*-Fe/N/C-800 | 83.6 | 49.5 | 2426 | 3.37 | 15.27 |
| *m*-Fe/N/C-900 | 86.1 | 61.2 | 2171 | 0.48 | 8.38 |
| *m*-Fe/N/C-950 | 88.8 | 145.6 | 4273 | 1.46 | 3.41 |

**Table 2S.** Comparison of this result with other reported references on the ORR activity.

| **Samples** | **E_onset_**  **(V *vs.* RHE)** | **E_1/2_**  **(V *vs.***  **RHE)** | **j_d_** | **References** |
| --- | --- | --- | --- | --- |
| **3D-Fe-N-C-700** | 0.92 | 0.83 | 4.95 mV cm^–2^ @ 0.60 vs. RHE | Carbon, 2017, 125, 640-648. |
| **3D-Co-N-C** | 1.00 | 0.83 | 5.90 mA cm^–2^ @ 0.65 vs. RHE | J. Mater. Chem. A, 2018, 6, 13050. |
| **Fe/N/APC-900** | 0.96 | 0.88 | 6.30 mV cm^–2^ @ 0.60 vs. RHE | J. Am. Chem. Soc., 2016, 138(10), 3570. |
| **Co-N*_x_*@CNF700** | 0.94 | 0.81 | 5.50 mA cm^–2^ @ 0.65 vs. RHE | Nature Commun., 2015, 6, 7343. |
| **FeNC-900** | 0.97 | 0.84 | 5.21 mA cm^–2^ @ 0.50 vs. RHE | Chem. Commun., 2018, 54, 1307. |
| **Fe-PANI@GD-900** | 0.96 | 0.82 | 4.50 mA cm^–2^ @ 0.50 vs. RHE | Carbon, 2017, 119, 201-210. |
| **FeN/C-PANI** | 0.95 | 0.83 | 5.30 mV cm^–2^ @ 0.60 vs. RHE | J.Alloys Comp., 2016, 686, 874. |
| **Co-N*_x_*@CNF700** | 0.94 | 0.81 | 5.50 mA cm^–2^ @ 0.65 vs. RHE | J. Am. Chem. Soc., 2014, 136(31), 10882. |
| **CoN/C-600** | 0.91 | 0.85 | 5.70 mA cm^–2^ @ 0.55 V *vs.*RHE | Inter. J. Hydrogen Energy, 2016, 41, 12995. |
| **Co-N*_x_*@CNF700** | 0.941 | 0.814 | 5.50 mA cm^–2^ @ 0.65 *vs.* RHE | J. Power Sources, 2018, 380, 174-184. |
| **ZnN*_x_*/BP** | 1.0 | 0.825 | 6.0 mA cm^–2^ @ -0.60V *vs.* SCE | Adv. Funct. Mater., 2017, 1700802. |
| ***m*-Fe/N/C-900** | **1.0** | **0.841** | **5.80 mA cm^–2^ @ 0.60 vs. RHE** | **This work** |
